# 1-Chloro­acetyl-2,6-bis­(3-fluoro­phen­yl)piperidin-4-one

**DOI:** 10.1107/S1600536809028529

**Published:** 2009-07-25

**Authors:** G. Aridoss, D. Gayathri, D. Velmurugan, M. S. Kim, Yeon Tae Jeong

**Affiliations:** a Division of Image Science and Information Engineering, Pukyong National University, Busan 608-739, Republic of Korea; b Centre of Advanced Study in Crystallography and Biophysics, University of Madras, Guindy Campus, Chennai 600 025, India

## Abstract

In the title compound C_19_H_16_ClF_2_NO_2_, the piperidone ring adopts a twist-boat conformation with the two out-of-plane atoms deviating by 0.544 (1) and 0.511 (1) Å from the plane through the remaining atoms in the ring. Sterically favoured non-H-atom C⋯O inter­molecular contacts are observed in the structure, within a 3.00 Å range. The crystal packing is stabilized by C—H⋯O and C—H⋯F hydrogen bonds and an inter­molecular π–π inter­action [centroid-centroid separation of 3.783 (1) Å]. Alternating C—H⋯O and C—H⋯F inter­molecular inter­actions generate chains running along the *a* axis, while a centrosymmetric *R*_2_^2^(16) ring involving C—H⋯O inter­actions is formed centred at (1/2, 1/2, 0).

## Related literature

For background to the biological activity of piperidines and piperidones and their derivatives, see: Richardo *et al.* (1979 ▶); Schneider (1996 ▶); Mukhtar & Wright (2005 ▶); Fleet *et al.* (1990 ▶); Winkler & Holan (1989 ▶); Aridoss *et al.* (2007*a* ▶, 2008 ▶, 2009*a* ▶). For related structures, see: Gayathri *et al.* (2008 ▶); Ramachandran *et al.* (2008 ▶); Aridoss *et al.* (2009*b* ▶). For the synthesis and stereochemistry, see: Krishnapillay *et al.* (2000 ▶); Aridoss *et al.* (2007*b* ▶). For ring conformational analysis, see: Cremer & Pople (1975 ▶); Nardelli (1983 ▶). 
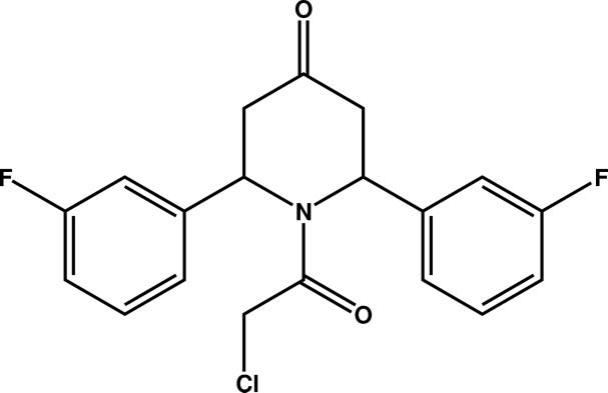


## Experimental

### 

#### Crystal data

C_19_H_16_ClF_2_NO_2_
*M* *_r_* = 363.78
Monoclinic, 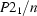

*a* = 10.7026 (8) Å
*b* = 8.2017 (6) Å
*c* = 19.0447 (15) Å
β = 100.629 (1)°
*V* = 1643.1 (2) Å^3^
*Z* = 4
Mo *K*α radiation
μ = 0.27 mm^−1^
*T* = 293 K
0.29 × 0.25 × 0.22 mm

#### Data collection

Bruker SMART APEX diffractometer
Absorption correction: none
18143 measured reflections
3875 independent reflections
3515 reflections with *I* > 2σ(*I*)
*R*_int_ = 0.018

#### Refinement

*R*[*F* ^2^ > 2σ(*F* ^2^)] = 0.039
*wR*(*F* ^2^) = 0.111
*S* = 1.03
3875 reflections
226 parameters
H-atom parameters constrained
Δρ_max_ = 0.34 e Å^−3^
Δρ_min_ = −0.35 e Å^−3^

### 

Data collection: *SMART* (Bruker, 2001 ▶); cell refinement: *SAINT* (Bruker, 2001 ▶); data reduction: *SAINT*; program(s) used to solve structure: *SHELXS97* (Sheldrick, 2008 ▶); program(s) used to refine structure: *SHELXL97* (Sheldrick, 2008 ▶); molecular graphics: *PLATON* (Spek, 2009 ▶); software used to prepare material for publication: *SHELXL97* and *PARST* (Nardelli, 1995 ▶).

## Supplementary Material

Crystal structure: contains datablocks I, global. DOI: 10.1107/S1600536809028529/bg2281sup1.cif

Structure factors: contains datablocks I. DOI: 10.1107/S1600536809028529/bg2281Isup2.hkl

Additional supplementary materials:  crystallographic information; 3D view; checkCIF report

## Figures and Tables

**Table 1:** Hydrogen-bond geometry (Å, °)

*D*—H⋯*A*	*D*—H	H⋯*A*	*D*⋯*A*	*D*—H⋯*A*
C2—H2*B*⋯O1^i^	0.97	2.60	3.415 (2)	142
C7—H7*A*⋯F2^ii^	0.97	2.49	3.270 (2)	137
C18—H18⋯O2^iii^	0.93	2.55	3.308 (2)	139

Symmetry codes: (i) 
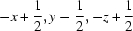
; (ii) 
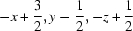
; (iii) 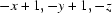.

**Fig. 1.**
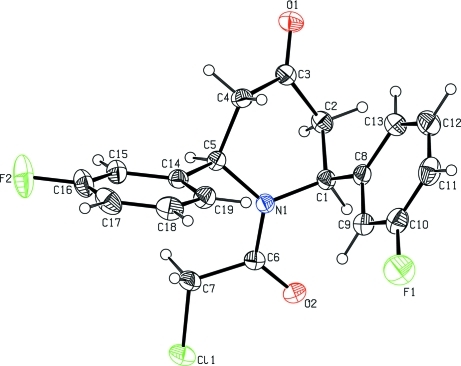
The molecular structure of the title compound(I), showing 30% probability displacement ellipsoids.

**Fig. 2.**
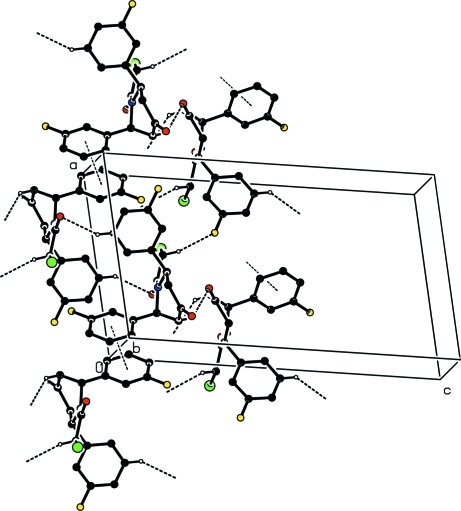
The molecular packing of (I), viewed approximately along the b axis, showing C—H···O and C—H···F hydrogen bonds, drawn as thick dashed lines, and π–π intermolecular interaction, drawn as narrow dashed lines. For clarity, hydrogen atoms which are not involved in hydrogen bonding are omitted.

## supplementary crystallographic information

### Comment

Amides are a prominent functional group in chemistry due to their being an integral part in biologically important polymers such as peptides and proteins. Functionalized piperidines are among the most common building blocks in natural products, and, more interestingly, in many biologically active compounds such as anopterine, pergoline, scopolamine and morphine (Richardo *et al.*, 1979, Schneider, 1996, Mukhtar & Wright, 2005). Piperidones also have high impact in medicinal field owing to their role as key chiral intermediates for the preparation of a variety of natural, synthetic and semi-synthetic pharmacophores with marked anticancer (Fleet *et al.*, 1990) and anti-HIV activities (Winkler & Holan, 1989). Particularly, amides derived from 2,6-diarylpiperidin-4-ones exhibited marked antibacterial and antitubercular activities (Aridoss *et al.* 2007*a*, 2008, 2009*a*). As a corollary of these interesting biological and pharmaceutical properties and synthetic utility, substantial interest has been demonstrated towards 2,6-diarylpiperidin-4-ones (Krishnapillay *et al.*, 2000, Aridoss *et al.*, 2007*b*). Recently, we have disclosed the crystal structures of variously substituted 2,6-diarylpiperidin-4-ones and their derivatives (Gayathri *et al.* 2008, Ramachandran *et al.*, 2008, Aridoss *et al.*, 2009*b*). In the interest of above, crystal structure of 1-chloroacetyl-2,6-bis (3-fluorophenyl)-piperidin-4-one is reported here.

The molecular structure of compound (I) is illustrated in Fig.1. The sum of the angles at N1 (357.3 (3)°) is in accordance with *sp*^2^ hybridization. The dihedral angle between the two phenyl rings is 65.8 (1)°. Fluorine atoms (F1 and F2) lie above the plane of the phenyl rings to which they are attached by 0.041 (1) and 0.003 (2) Å, respectively. The torsion angle around O2—C6—C7—Cl1 indicates the planarity of chloroacetyl moiety. In the present structure, the piperidone ring adops a twist-boat conformation with atoms C1 and C4 deviating by 0.544 (1) and 0.511 (1) Å, respectively, from the least-sqaures plane defined by the remaining atoms (N1/C2/C3/C5) in the ring. When compared with the reported structures of piperidone derivatives (Gayathri *et al.*, 2007, Ramachandran *et al.*, 2008, Aridoss *et al.*, 2009*b*), it is clear that the conformation of the piperidone ring is highly influenced by the substitutions at various positions. The puckering parameters (Cremer & Pople, 1975) and the smallest displacement asymmetry parameters (Nardelli, 1983) for piperidone ring are q_2_ = 0.671 (1) Å, q_3_ = 0.045 (1) Å; Q_T_ = 0.672 (1)Å and θ = 86.0 (1)°, respectively.

Sterically favoured short non-hydrogen intermolecular contacts are observed between O1 and C6 (-*x* + 1/2, *y* + 1/2, -*z* + 1/2) and C6 and O1 (-*x* + 1/2, *y* - 1/2, -*z* + 1/2), each within a distance of 2.98 (2) Å. The crystal packing is stabilized by C—H···O and C—H···F hydrogen bonds and a π–π intermolecular interaction. Atoms C2 and C7 act as donors to O1 (-*x* + 1/2, *y* - 1/2, -*z* + 1/2) and F2 (-*x* + 3/2, *y* - 1/2, -*z* + 1/2) generating a chain running along the *a* axis. Atom C18 acts as a donor to O2 (-*x* + 1, -*y* + 1, -*z*) generating a centrosymmetric dimer of *R*_2_^2^(16) ring centred at (1/2, 1/2, 0). An intermolecular π–π interaction is observed between the symmetry related six-membered ring, *Cg*···*Cg*^i^ [symmetry code: (i) -*x*, 1 - *y*, -*z*; *Cg* is the centroid of the C8—C13 ring], with the centroid-centroid separation of 3.783 (1)Å and with the slippage of 1.191 Å.

### Experimental

The title compound was obtained by adopting our earlier method (Aridoss *et al.* 2007*a*) with slight modification. To a solution of 2,6-bis(3-fluorophenyl)- piperidin-4-one (1 equiv.) and NEt_3_ (1.5 equiv.) in freshly distilled benzene, chloroacetyl chloride (1 equiv.) in benzene was added drop wise. Stirring was continued until the completion of reaction. Later, it was poured into water and extracted with ethyl acetate. The combined organic extract was then washed well with a 3% sodium bicarbonate solution, brined and dried over anhydrous sodium sulfate. This, upon evaporation and subsequent recrystallization of the title compound in distilled ethanol afforded fine crystals suitable for X-ray diffraction study.

### Refinement

All H-atoms were positioned geometrically and refined using a riding model, with d(C—H) = 0.93 Å, *U*_iso_ = 1.2*U*_eq_ (C) for aromatic, 0.97 Å, *U*_iso_ = 1.2*U*_eq_ (C) for CH_2_ and 0.98 Å, *U*_iso_ = 1.2*U*_eq_ (C) for CH atoms.

### Crystal data

**Table:**

C_19_H_16_ClF_2_NO_2_	*F*(000) = 752
*M**_r_* = 363.78	*D*_x_ = 1.471 Mg m^−^^3^
Monoclinic, *P*2_1_/*n*	Mo *K*α radiation, λ = 0.71073 Å
Hall symbol: -P 2yn	Cell parameters from 2558 reflections
*a* = 10.7026 (8) Å	θ = 2.0–28.1°
*b* = 8.2017 (6) Å	µ = 0.27 mm^−^^1^
*c* = 19.0447 (15) Å	*T* = 293 K
β = 100.629 (1)°	Block, colorless
*V* = 1643.1 (2) Å^3^	0.29 × 0.25 × 0.22 mm
*Z* = 4	

### Data collection

**Table:**

Bruker SMART APEX diffractometer	3515 reflections with *I* > 2σ(*I*)
Radiation source: fine-focus sealed tube	*R*_int_ = 0.018
graphite	θ_max_ = 28.1°, θ_min_ = 2.0°
ω scans	*h* = −14→13
18143 measured reflections	*k* = −10→10
3875 independent reflections	*l* = −25→24

### Refinement

**Table:**

Refinement on *F*^2^	Primary atom site location: structure-invariant direct methods
Least-squares matrix: full	Secondary atom site location: difference Fourier map
*R*[*F*^2^ > 2σ(*F*^2^)] = 0.039	Hydrogen site location: inferred from neighbouring sites
*wR*(*F*^2^) = 0.111	H-atom parameters constrained
*S* = 1.03	*w* = 1/[σ^2^(*F*_o_^2^) + (0.0604*P*)^2^ + 0.4707*P*] where *P* = (*F*_o_^2^ + 2*F*_c_^2^)/3
3875 reflections	(Δ/σ)_max_ < 0.001
226 parameters	Δρ_max_ = 0.34 e Å^−^^3^
0 restraints	Δρ_min_ = −0.35 e Å^−^^3^

### Special details

**Table:**

Geometry. All e.s.d.'s (except the e.s.d. in the dihedral angle between two l.s. planes) are estimated using the full covariance matrix. The cell e.s.d.'s are taken into account individually in the estimation of e.s.d.'s in distances, angles and torsion angles; correlations between e.s.d.'s in cell parameters are only used when they are defined by crystal symmetry. An approximate (isotropic) treatment of cell e.s.d.'s is used for estimating e.s.d.'s involving l.s. planes.
Refinement. Refinement of *F*^2^ against ALL reflections. The weighted *R*-factor *wR* and goodness of fit *S* are based on *F*^2^, conventional *R*-factors *R* are based on *F*, with *F* set to zero for negative *F*^2^. The threshold expression of *F*^2^ > σ(*F*^2^) is used only for calculating *R*-factors(gt) *etc*. and is not relevant to the choice of reflections for refinement. *R*-factors based on *F*^2^ are statistically about twice as large as those based on *F*, and *R*- factors based on ALL data will be even larger.

### Fractional atomic coordinates and isotropic or equivalent isotropic displacement parameters (Å^2^)

**Table:**

	*x*	*y*	*z*	*U*_iso_*/*U*_eq_	
C1	0.19220 (11)	0.60188 (15)	0.12209 (7)	0.0342 (3)	
H1	0.1633	0.4907	0.1291	0.041*	
C2	0.14537 (12)	0.70807 (17)	0.17787 (7)	0.0391 (3)	
H2A	0.0532	0.7101	0.1680	0.047*	
H2B	0.1725	0.6601	0.2248	0.047*	
C3	0.19437 (12)	0.87902 (16)	0.17849 (6)	0.0355 (3)	
C4	0.31441 (12)	0.89729 (15)	0.14859 (7)	0.0366 (3)	
H4A	0.3605	0.9917	0.1705	0.044*	
H4B	0.2914	0.9184	0.0977	0.044*	
C5	0.40356 (11)	0.74875 (14)	0.15997 (6)	0.0315 (2)	
H5	0.4393	0.7400	0.2111	0.038*	
C6	0.38663 (12)	0.44552 (15)	0.14846 (6)	0.0342 (3)	
C7	0.52903 (13)	0.44165 (16)	0.17627 (8)	0.0437 (3)	
H7A	0.5478	0.4980	0.2218	0.052*	
H7B	0.5722	0.4986	0.1430	0.052*	
C8	0.14016 (11)	0.64593 (15)	0.04440 (7)	0.0359 (3)	
C9	0.18650 (13)	0.56233 (18)	−0.00921 (8)	0.0438 (3)	
H9	0.2497	0.4839	0.0024	0.053*	
C10	0.13769 (15)	0.5973 (2)	−0.07922 (8)	0.0497 (3)	
C11	0.04478 (16)	0.7120 (2)	−0.09979 (8)	0.0530 (4)	
H11	0.0147	0.7346	−0.1478	0.064*	
C12	−0.00203 (16)	0.7923 (2)	−0.04692 (9)	0.0548 (4)	
H12	−0.0659	0.8695	−0.0593	0.066*	
C13	0.04442 (14)	0.76014 (18)	0.02456 (8)	0.0459 (3)	
H13	0.0112	0.8156	0.0596	0.055*	
C14	0.51171 (11)	0.78384 (15)	0.12008 (7)	0.0330 (2)	
C15	0.62680 (12)	0.84067 (18)	0.15766 (8)	0.0420 (3)	
H15	0.6415	0.8480	0.2072	0.050*	
C16	0.71890 (13)	0.8861 (2)	0.11948 (9)	0.0528 (4)	
C17	0.70255 (15)	0.8775 (2)	0.04699 (10)	0.0558 (4)	
H17	0.7667	0.9098	0.0231	0.067*	
C18	0.58841 (15)	0.8196 (2)	0.01009 (8)	0.0507 (4)	
H18	0.5751	0.8119	−0.0394	0.061*	
C19	0.49307 (14)	0.77279 (17)	0.04628 (7)	0.0416 (3)	
H19	0.4163	0.7338	0.0209	0.050*	
N1	0.33311 (9)	0.59708 (12)	0.13708 (5)	0.0316 (2)	
O1	0.14246 (10)	0.99449 (13)	0.20033 (6)	0.0477 (2)	
O2	0.32477 (10)	0.32101 (12)	0.13649 (6)	0.0467 (2)	
F1	0.18216 (12)	0.51239 (17)	−0.13076 (6)	0.0785 (3)	
F2	0.83117 (10)	0.9428 (2)	0.15588 (7)	0.0907 (4)	
Cl1	0.58658 (4)	0.24037 (5)	0.18722 (3)	0.06261 (15)	

### Atomic displacement parameters (Å^2^)

**Table:**

	*U*^11^	*U*^22^	*U*^33^	*U*^12^	*U*^13^	*U*^23^
C1	0.0292 (5)	0.0319 (6)	0.0411 (6)	−0.0020 (4)	0.0055 (5)	0.0025 (5)
C2	0.0355 (6)	0.0431 (7)	0.0411 (6)	0.0005 (5)	0.0129 (5)	0.0045 (5)
C3	0.0356 (6)	0.0407 (7)	0.0299 (5)	0.0040 (5)	0.0056 (4)	−0.0001 (5)
C4	0.0389 (6)	0.0302 (6)	0.0422 (6)	−0.0006 (5)	0.0118 (5)	−0.0019 (5)
C5	0.0318 (6)	0.0319 (6)	0.0309 (5)	−0.0024 (4)	0.0060 (4)	−0.0012 (4)
C6	0.0375 (6)	0.0327 (6)	0.0328 (5)	0.0023 (5)	0.0075 (5)	0.0006 (4)
C7	0.0392 (7)	0.0337 (6)	0.0572 (8)	0.0069 (5)	0.0060 (6)	0.0044 (6)
C8	0.0310 (6)	0.0328 (6)	0.0420 (6)	−0.0048 (5)	0.0020 (5)	−0.0005 (5)
C9	0.0399 (7)	0.0435 (7)	0.0458 (7)	0.0008 (5)	0.0021 (5)	−0.0040 (6)
C10	0.0496 (8)	0.0558 (9)	0.0434 (7)	−0.0096 (7)	0.0075 (6)	−0.0066 (6)
C11	0.0545 (8)	0.0570 (9)	0.0425 (7)	−0.0111 (7)	−0.0043 (6)	0.0087 (7)
C12	0.0507 (8)	0.0511 (8)	0.0571 (9)	0.0078 (7)	−0.0045 (7)	0.0090 (7)
C13	0.0414 (7)	0.0447 (8)	0.0495 (8)	0.0051 (6)	0.0026 (6)	0.0006 (6)
C14	0.0319 (6)	0.0297 (5)	0.0383 (6)	0.0006 (4)	0.0091 (5)	0.0008 (4)
C15	0.0356 (6)	0.0450 (7)	0.0443 (7)	−0.0028 (5)	0.0046 (5)	0.0046 (6)
C16	0.0303 (6)	0.0584 (9)	0.0685 (10)	−0.0043 (6)	0.0056 (6)	0.0149 (7)
C17	0.0423 (8)	0.0624 (10)	0.0686 (10)	0.0073 (7)	0.0261 (7)	0.0195 (8)
C18	0.0588 (9)	0.0532 (9)	0.0451 (7)	0.0056 (7)	0.0227 (7)	0.0028 (6)
C19	0.0437 (7)	0.0433 (7)	0.0388 (6)	−0.0024 (5)	0.0104 (5)	−0.0027 (5)
N1	0.0294 (5)	0.0295 (5)	0.0353 (5)	−0.0005 (4)	0.0041 (4)	0.0020 (4)
O1	0.0472 (5)	0.0474 (6)	0.0510 (6)	0.0082 (4)	0.0157 (4)	−0.0071 (4)
O2	0.0474 (5)	0.0311 (5)	0.0601 (6)	−0.0014 (4)	0.0060 (4)	−0.0032 (4)
F1	0.0865 (8)	0.0996 (9)	0.0501 (6)	0.0098 (7)	0.0145 (5)	−0.0162 (6)
F2	0.0411 (5)	0.1322 (12)	0.0927 (8)	−0.0331 (6)	−0.0035 (5)	0.0304 (8)
Cl1	0.0550 (2)	0.0405 (2)	0.0879 (3)	0.01621 (16)	0.0017 (2)	0.00367 (18)

### Geometric parameters (Å, °)

**Table:**

C1—N1	1.4827 (15)	C8—C9	1.3948 (19)
C1—C8	1.5251 (17)	C9—C10	1.370 (2)
C1—C2	1.5279 (18)	C9—H9	0.9300
C1—H1	0.9800	C10—F1	1.3590 (19)
C2—C3	1.4963 (19)	C10—C11	1.373 (2)
C2—H2A	0.9700	C11—C12	1.373 (3)
C2—H2B	0.9700	C11—H11	0.9300
C3—O1	1.2099 (16)	C12—C13	1.386 (2)
C3—C4	1.5056 (17)	C12—H12	0.9300
C4—C5	1.5379 (17)	C13—H13	0.9300
C4—H4A	0.9700	C14—C19	1.3857 (18)
C4—H4B	0.9700	C14—C15	1.3861 (18)
C5—N1	1.4781 (15)	C15—C16	1.380 (2)
C5—C14	1.5246 (16)	C15—H15	0.9300
C5—H5	0.9800	C16—F2	1.3542 (18)
C6—O2	1.2154 (16)	C16—C17	1.361 (2)
C6—N1	1.3691 (15)	C17—C18	1.376 (2)
C6—C7	1.5187 (18)	C17—H17	0.9300
C7—Cl1	1.7609 (14)	C18—C19	1.387 (2)
C7—H7A	0.9700	C18—H18	0.9300
C7—H7B	0.9700	C19—H19	0.9300
C8—C13	1.3883 (19)		
			
N1—C1—C8	111.69 (10)	C9—C8—C1	118.56 (12)
N1—C1—C2	109.49 (10)	C10—C9—C8	119.11 (14)
C8—C1—C2	115.56 (11)	C10—C9—H9	120.4
N1—C1—H1	106.5	C8—C9—H9	120.4
C8—C1—H1	106.5	F1—C10—C9	118.32 (15)
C2—C1—H1	106.5	F1—C10—C11	118.48 (14)
C3—C2—C1	112.34 (10)	C9—C10—C11	123.19 (15)
C3—C2—H2A	109.1	C12—C11—C10	117.56 (14)
C1—C2—H2A	109.1	C12—C11—H11	121.2
C3—C2—H2B	109.1	C10—C11—H11	121.2
C1—C2—H2B	109.1	C11—C12—C13	121.06 (15)
H2A—C2—H2B	107.9	C11—C12—H12	119.5
O1—C3—C2	123.62 (12)	C13—C12—H12	119.5
O1—C3—C4	122.01 (12)	C12—C13—C8	120.59 (15)
C2—C3—C4	114.36 (10)	C12—C13—H13	119.7
C3—C4—C5	114.73 (10)	C8—C13—H13	119.7
C3—C4—H4A	108.6	C19—C14—C15	119.59 (12)
C5—C4—H4A	108.6	C19—C14—C5	120.83 (11)
C3—C4—H4B	108.6	C15—C14—C5	119.36 (11)
C5—C4—H4B	108.6	C16—C15—C14	118.16 (13)
H4A—C4—H4B	107.6	C16—C15—H15	120.9
N1—C5—C14	113.88 (9)	C14—C15—H15	120.9
N1—C5—C4	110.83 (10)	F2—C16—C17	118.22 (14)
C14—C5—C4	106.77 (9)	F2—C16—C15	118.41 (15)
N1—C5—H5	108.4	C17—C16—C15	123.37 (14)
C14—C5—H5	108.4	C16—C17—C18	118.13 (13)
C4—C5—H5	108.4	C16—C17—H17	120.9
O2—C6—N1	122.39 (11)	C18—C17—H17	120.9
O2—C6—C7	121.65 (11)	C17—C18—C19	120.47 (14)
N1—C6—C7	115.96 (11)	C17—C18—H18	119.8
C6—C7—Cl1	111.56 (10)	C19—C18—H18	119.8
C6—C7—H7A	109.3	C14—C19—C18	120.29 (14)
Cl1—C7—H7A	109.3	C14—C19—H19	119.9
C6—C7—H7B	109.3	C18—C19—H19	119.9
Cl1—C7—H7B	109.3	C6—N1—C5	122.80 (10)
H7A—C7—H7B	108.0	C6—N1—C1	115.82 (10)
C13—C8—C9	118.47 (13)	C5—N1—C1	118.67 (9)
C13—C8—C1	122.89 (12)		
			
N1—C1—C2—C3	59.23 (13)	C4—C5—C14—C19	−75.31 (14)
C8—C1—C2—C3	−67.92 (14)	N1—C5—C14—C15	−138.10 (12)
C1—C2—C3—O1	156.26 (12)	C4—C5—C14—C15	99.24 (13)
C1—C2—C3—C4	−23.06 (15)	C19—C14—C15—C16	0.6 (2)
O1—C3—C4—C5	149.28 (12)	C5—C14—C15—C16	−173.98 (13)
C2—C3—C4—C5	−31.38 (15)	C14—C15—C16—F2	179.52 (14)
C3—C4—C5—N1	50.02 (14)	C14—C15—C16—C17	−0.2 (2)
C3—C4—C5—C14	174.56 (10)	F2—C16—C17—C18	179.94 (16)
O2—C6—C7—Cl1	0.30 (17)	C15—C16—C17—C18	−0.4 (3)
N1—C6—C7—Cl1	−179.00 (9)	C16—C17—C18—C19	0.4 (2)
N1—C1—C8—C13	−134.11 (13)	C15—C14—C19—C18	−0.6 (2)
C2—C1—C8—C13	−8.06 (18)	C5—C14—C19—C18	173.95 (13)
N1—C1—C8—C9	49.02 (15)	C17—C18—C19—C14	0.0 (2)
C2—C1—C8—C9	175.06 (11)	O2—C6—N1—C5	171.94 (12)
C13—C8—C9—C10	1.0 (2)	C7—C6—N1—C5	−8.77 (16)
C1—C8—C9—C10	178.01 (12)	O2—C6—N1—C1	10.87 (17)
C8—C9—C10—F1	−178.72 (13)	C7—C6—N1—C1	−169.84 (11)
C8—C9—C10—C11	0.3 (2)	C14—C5—N1—C6	66.79 (14)
F1—C10—C11—C12	177.74 (15)	C4—C5—N1—C6	−172.80 (10)
C9—C10—C11—C12	−1.2 (2)	C14—C5—N1—C1	−132.65 (11)
C10—C11—C12—C13	1.0 (3)	C4—C5—N1—C1	−12.24 (14)
C11—C12—C13—C8	0.3 (3)	C8—C1—N1—C6	−109.19 (12)
C9—C8—C13—C12	−1.3 (2)	C2—C1—N1—C6	121.50 (11)
C1—C8—C13—C12	−178.13 (13)	C8—C1—N1—C5	88.92 (13)
N1—C5—C14—C19	47.35 (16)	C2—C1—N1—C5	−40.39 (14)

### Hydrogen-bond geometry (Å, °)

**Table:**

*D*—H···*A*	*D*—H	H···*A*	*D*···*A*	*D*—H···*A*
C2—H2B···O1^i^	0.97	2.60	3.415 (2)	142
C7—H7A···F2^ii^	0.97	2.49	3.270 (2)	137
C18—H18···O2^iii^	0.93	2.55	3.308 (2)	139

Symmetry codes: (i) −*x*+1/2, *y*−1/2, −*z*+1/2; (ii) −*x*+3/2, *y*−1/2, −*z*+1/2; (iii) −*x*+1, −*y*+1, −*z*.
